# Neurological soft signs and brain morphology in people living with HIV

**DOI:** 10.1007/s13365-022-01071-6

**Published:** 2022-03-29

**Authors:** Christina J. Herold, Li Kong, María Elena Ceballos, Johannes Schröder, Pablo Toro

**Affiliations:** 1 Section of Geriatric Psychiatry, Department of General Psychiatry, University of Heidelberg, Heidelberg, Germany; 2 Department of Psychology, Shanghai Normal University, Shanghai, China; 3 Department of Infectious Diseases, Medicine School, Pontificia Universidad Católica de Chile, Santiago, Chile; 4 Department of Psychiatry, Medicine School, Pontificia Universidad Católica de Chile, Santiago, Chile

**Keywords:** NSS, HIV-associated neurocognitive disorder, HAND, HIV-associated dementia, Structural MRI

## Abstract

Neurological soft signs (NSS) are a common feature of severe psychiatric disorders such as schizophrenia but are also prevalent in organic brain diseases like HIV-associated neurocognitive disorder (HAND) or Alzheimer’s disease. While distinct associations between NSS, neurocognition, and cerebral regions were demonstrated in schizophrenia, these associations still have to be elucidated in HIV. Therefore, we investigated 36 persons with HIV of whom 16 were neurocognitively healthy and 20 were diagnosed with HAND. NSS were assessed using the Heidelberg scale. NSS scores were correlated with gray matter (GM) using whole brain voxel-based morphometry. Results showed significantly elevated NSS in the HAND group when compared to the neurocognitively healthy with respect to NSS total score and the subscores “orientation” and “complex motor tasks”. While the two groups showed only minor, non-significant GM differences, higher NSS scores (subscales “motor coordination”, “orientation”) were significantly correlated with GM reduction in the right insula and cerebellum (FWE-corrected). Our results corroborate elevated NSS in HIV+ patients with HAND in contrast to cognitively unimpaired patients. In addition, cerebral correlates of NSS with GM reductions in insula and cerebellum were revealed. Taken together, NSS in this patient group could be considered a marker of cerebral damage and neurocognitive deficits.

## Introduction

In the era of potent antiretroviral therapy (ART), HIV-associated dementia rarely develops, while in contrast, milder forms of HIV-associated neurocognitive disorder (HAND) increase in prevalence (Brew and Chan 2014; Clifford and Ances 2013; Heaton et al. 2015). A total of 30–50% of patients with HIV demonstrate HAND (Mishra and Seth 2012; Saloner and Cysique 2017; Smith and Sacktor 2014). According to the National Institute of Mental Health and the National Institute of Neurological Diseases and Stroke, three categories depending on the severity of the disorder can be differentiated and assessed clinically and by neuropsychological testing: Asymptomatic neurocognitive impairment (ANI) refers to cognitive impairment of 1 standard deviation below the mean in two cognitive domains with intact activities of daily living. Mild neurocognitive disorder (MND) means a deficit of 1 standard deviation in at least two cognitive domains with impaired activities of daily living. HIV-associated dementia is defined by a cognitive impairment of 2 standard deviations below the mean in at least two cognitive domains with a marked impairment of activities of daily living (Antinori et al. 2007; Clifford and Ances 2013; Heaton et al. 2015).

Cerebral changes associated with HIV infection involve gray matter (GM) atrophy, white matter lesions, and ventricular expansion. More specifically, one of the most common findings is a reduction of basal ganglia volume (particularly caudate nucleus), which is clearly associated with cognitive impairment and motor dysfunction; similar findings have been reported for white matter atrophy. These findings especially apply to patients with advanced HIV disease including HIV-associated dementia (Arendt 2020; Brew and Chan 2014; Masters and Ances 2014; Paul et al. 2002; Tate et al. 2011; Woods et al. 2009).

Neurological soft signs (NSS) are minor motor and sensory deficits, which are established in patients with major psychiatric disorders such as schizophrenia or bipolar disorder (Chrobak et al. 2016; Heinrichs and Buchanan 1988; Schröder et al. 1992; Zhao et al. 2013). However, NSS can also be demonstrated in patients with organic brain changes such as HAND, mild cognitive impairment (MCI), or Alzheimer’s disease (AD) (Li et al. 2012; Seidl et al. 2009; Toro et al. 2018; Urbanowitsch et al. 2015). That NSS are related to neurocognitive functioning was demonstrated in patients with chronic schizophrenia (Herold et al. 2019), bipolar disorder (Goswami et al. 2006), AD (Seidl et al. 2009; Urbanowitsch et al. 2015), and HAND (Forno et al. 2020; Toro et al. 2018) with the latter studies reporting elevated levels of NSS with more severe forms of dementia in contrast to precursor states. Hence, NSS can be defined as a transdiagnostic phenomenon which corresponds to psychopathological symptoms, cognitive deficits, and underlying brain changes in any severe psychiatric disorder (Schröder and Herold 2021).

Subtle motor dysfunction similar to NSS was described in patients with HIV infection already in the 1990s (Arendt et al. 1992, 1990, 1994). Morphologically, their motor test performance was similar to that of patients with manifest basal ganglia disease, while MRI scans of all patients showed no pathological findings. The previous finding of motor abnormalities^1^ in 50% of HIV-infected patients in asymptomatic clinical stage (and in 80% of the patients in more advanced clinical stages) (Arendt et al. 1990) was confirmed and extended by Valcour et al. (2008). The examination of 229 HIV+ participants using the motor exam of the Unified Parkinson’s Disease Rating Scale^2^ for the assessment of extrapyramidal motor signs revealed higher scores with increasing age (20–40 vs. > 50 years) and depending on HIV status: three or more signs were found in 40.7% of patients with HIV in comparison to 15.7% of the controls. Moreover, motor scores increased with worsening of cognitive category (normal cognition < minor cognitive motor disorder < HIV-associated dementia). These results are consistent with HIV-specific neuropathology in subcortical and deep gray matter structures that support motor functions and underline the importance of motor signs in this patient group, especially given the increasing age of people living with HIV (Eggers et al. 2017). These findings were finally conceptualized by Robinson-Papp et al. (2008) with the HIV-Dementia Motor Scale^3^ that captures motor abnormalities which are associated with cognitive impairment in HIV (Robinson-Papp et al. 2008).

Another study recently showed impairments of motor performance while multitasking in a small group of elderly (60 ± 8 years) HIV+ subjects (*N* = 25 vs. 22 healthy controls), while cognitive performance was spared (Kronemer et al. 2017). Recently, it has been reported that 69% of patients with HIV showed motor abnormalities^4^; 27% were classified as severe (*N* = 354, 60 ± 9 years). The most common abnormalities were registered in gait (54%) and coordination (39%) and were associated with HAND/cognitive impairment (Robinson-Papp et al. 2020).

Elevated NSS have been described in both ANI and MND, but not HIV+ patients without neurocognitive deficits (Toro et al. 2018). Recently, these findings were confirmed and extended by the same group with significant correlations between increased NSS scores and cognitive impairments, i.e., in episodic memory and executive functions (Forno et al. 2020). Furthermore, the authors demonstrated that NSS total score is an important predictor of prefrontal and hippocampal function, which has been shown to be affected in patients with HIV (Castelo et al. 2006; Maki et al. 2009).

In patients with schizophrenia, neuroimaging studies identified changes in sensorimotor cortices, supplementary motor area, basal ganglia, thalamus, and cerebellum as cerebral correlates of NSS (Hirjak et al. 2015; Zhao et al. 2014). These results suggest that NSS in schizophrenia refer to disseminated cerebral alterations, which involve a large cerebello-thalamo-prefrontal network (Zhao et al. 2014) rather than to focal deficits in discrete “motor” sites. Along with this, Zhou et al. (2017) recently showed a spatially coincident GM reduction and abnormal activation in bilateral posterior insula during motor performance in a group of patients with HIV.

Given the transdiagnostic character of NSS, one may assume that similar patterns of cerebral associations also apply to other neuropsychiatric disorders, such as HIV infection, which lead to increased NSS levels. To test this hypothesis in a clinical study, we investigated NSS with respect to structural cerebral alterations in HIV+ patients without any evidence of neurocognitive deficits and in HIV+ patients with HAND. According to the given literature, we expected a confirmation of higher NSS levels in patients with HAND in contrast to cognitively healthy patients and significant correlations of NSS scores with reduced GM in cortical and subcortical sites.

## Methods

### Patients

The data were taken from a previous study of our group (Toro et al. 2018; *N* = 67, 40 ± 10 years of age); however, only data from patients with HIV infection who received a structural magnetic resonance imaging (MRI) of the brain were drawn upon. All patients were recruited from an outpatient infectious disease clinic (Red Salud UC-CHRISTUS).

A sample of 36 right-handed male patients with HIV infection was examined, with a mean age of 38.4 years (SD = 10.0 years) and a mean education of 15.6 years (2.4 years). These parameters were similar and without significant differences between the subgroup presented here and the whole group of Toro et al. (2018); the same applies to the NSS scores (*p* > 0.20).

Clinical psychiatric disorders were diagnosed using the Spanish version of the Structured Clinical Interview for the DSM-4 TR (First et al. 2002). While none of the patients had a history of neurological or other severe physical conditions, 18 patients had a lifetime history of major depressive disorder. Eight were diagnosed with current depression, while five patients had substance abuse in the past.

A physical and neurological exam was performed by an infectious disease specialist. Modern ART was consigned, and lymphocyte CD4 count and HIV viral load were registered.

For neuropsychological assessment, the Cambridge Neuropsychological Test Automated Battery was applied (CANTAB; Fray and Robbins 1996). In addition, phonemic and semantic verbal fluency was examined (Chilean norms: Guàrdia-Olmos et al. 2015). *Z*-values corrected for age and sex were obtained using CANTAB normative data. The verbal IQ for the normalization was obtained using verbal scale of the WAIS, validated for the Chilean population (Rosas et al. 2014). All neuropsychological tests were performed by two trained clinical psychologists. Sixteen of the patients were neurocognitively healthy, 8 were diagnosed with ANI, and 12 were suffering from MND, according to the criteria defined by the National Institute of Mental Health and the National Institute of Neurological Diseases and Stroke (Antinori et al. 2007; Clifford and Ances 2013). Clinical diagnoses were established by a consensus of an infectious disease specialist (M.E.C.) and a psychiatrist (P.T.).

The investigations were approved by the ethics committee of the Pontificia Universidad Católica de Chile (project number 12–119). Written informed consent was obtained from all participants after the procedures of the study had been fully explained in accordance with the Declaration of Helsinki.

### NSS assessment

NSS were examined using the Heidelberg Scale^5^ (Schröder et al. 1992, 1993), which consists of five items assessing “motor coordination” (Ozeretzki’s test, diadochokinesis, pronation/supination, finger-to-thumb opposition, speech articulation), three items assessing “integrative functions” (gait, tandem walking, two-point discrimination), two items assessing “complex motor tasks” (finger-to-nose test, fist-edge-palm test), four items assessing “right/left and spatial orientation” (right/left orientation, graphesthesia, face-hand test, stereognosis), and two items assessing “hard signs” (arm holding test, mirror movements). Ratings are given on a 0 (no prevalence) to 3 (marked prevalence) point scale.

A sufficient internal reliability (Cronbach’s alpha = 0.85/0.89 in patients with schizophrenia/healthy controls), retest reliability (*r* = 0.80), and interrater reliability (*r* = 0.88 in patients with schizophrenia; *r* = 0.87–0.92 in healthy controls) of the Heidelberg NSS scale were established in previous studies (Bachmann et al. 2005; Schröder et al. 1992; Valenzuela et al. 2014). The NSS scale was administered by a clinical psychiatrist (P.T.) and NSS were examined prior to cognitive testing to guarantee an assessment blind to cognitive condition.

### Imaging data acquisition and voxel-based morphometry

The MRI T1-weighted data were obtained at the Medicine School of the Pontificia Universidad Católica de Chile with a 1.5-Tesla scanner (Philipps). The following acquisition parameters were used: T1-FFE, 175 sagittal slices, voxel size = 1.0 × 1.0 × 1.0 mm, image matrix = 240 × 240, flip angle 30°, *TR* = 25, *TE* = 4.60.

After all images were visually screened for artifacts, the origin was manually set at the anterior commissure. Statistical Parametric Mapping software^6^ (SPM12, v6685) implemented within MATLAB R2015a was used for voxel-based morphometry (VBM) analyses by applying the computational anatomy toolbox^7^ (CAT12.5 r963) with default parameters. As a last step, the images were smoothed with an 8-mm full width at half maximum (FWHM) Gaussian kernel.

### Statistical analysis

For group comparisons (neurocognitively healthy, *N* = 16 vs. impaired, *N* = 20), the subgroups of patients with ANI (*N* = 8) and MND (*N* = 12) were merged into a single group with HAND. *P-*values of less than 0.05 were considered significant; all computations were performed using SPSS 23. Clinical variables were compared between groups by calculating independent two-tailed two-sample *t*-tests or *χ*^2^ tests, respectively. Group differences between cognitively unimpaired and HAND patients with respect to GM were performed with two-sample *t*-test, TIV (total intracranial volume) was introduced as a covariate. Pearson’s correlation coefficients (Pearson’s *r*) were calculated to explore potential associations between NSS scores and age, education, or absolute GM volumes, respectively.

Voxel-wise regression analyses (*N* = 36) were calculated to evaluate the relationships between GM and NSS scores while using TIV as a covariate, orthogonality of the respective variables was assured. The resulting T-maps were thresholded for a significance level of *p* < 0.001 uncorrected with an extent threshold of *k* = 100 voxels. In a second step, the results were corrected for multiple comparisons (family-wise error, FWE) at cluster level, and *p-*values < 0.05 were considered significant.

The coordinates were converted to Talairach space by using the icbm2tal transform (Laird et al. 2010; Lancaster et al. 2007) implemented within GingerALE 3.0.2^8^ (Eickhoff et al. 2012, 2009; Turkeltaub et al. 2012), Talairach labels were then generated via Talairach Client 2.4.3^9^ (Lancaster et al. 1997, 2000).

## Results

### Demographic and clinical characteristics

Clinical characteristics of the whole patient group and the subgroups “cognitively unimpaired” and “patients with HAND” are summarized in Table 1. Diagnostic groups differed not significantly with respect to any clinical variable. The majority of patients were on ART with a high percentage with undetectable actual viral load.

**Table 1:** Clinical characteristics of the whole patient group and the respective subgroups, comparisons: cognitively unimpaired patients vs. cognitively impaired/HAND patients

	**Patients, whole group** **(*N* = 36)**	**Subgroups**		**Main effects, *T*-values**_**[df]**_
		Cognitively unimpaired patients (*N* = 16)	HAND patients (*N* = 20)	
Age (years)	38.44 (10.00)	37.81 (8.46)	38.95 (11.27)	*T*_[1, 34]_ = −0.335; *p* = 0.740
Education (years)	15.64 (2.39)	15.63 (2.25)	15.65 (2.56)	*T*_[1, 34]_ = −0.031; *p* = 0.976
Depression, lifetime/current, *N* (%)	18 (50.0)/8 (22.2)	8 (50.0)/3 (18.8)	10 (50.0)/5 (25.0)	*χ*^*2*^ = 0.281; *p* = 0.869
Lifetime substance abuse, *N* (%)	5 (13.9)	2 (13.3)	3 (17.6)	*χ*^*2*^ = 0.112; *p* = 0.737
On ART, *N* (%)	33 (91.7)	15 (93.8)	18 (90.0)	*χ*^*2*^ = 0.164; *p* = 0.686
Actual CD4 count (cells/mm^3^)	447.25 (193.00)	413.56 (188.44)	474.20 (197.14)	*T*_[1, 34]_ = −0.935; *p* = 0.356
CD4 nadir (cells/mm^3^)	221.89 (150.94)	193.94 (140.54)	245.42 (159.05)	*T*_[1, 33]_ = −1.005; *p* = 0.322
Undetectable actual viral load (< 20 copies/mL), *N* (%)	26 (72.2)	13 (81.3)	13 (65.0)	*χ*^*2*^ = 1.170; *p* = 0.279
Viral load (copies/mL) at time of diagnosis	236543.68 (392909.41)	244909.33 (486634.69)	228700.88 (295963.26)	*T*_[1, 29]_ = 0.113; *p* = 0.911

Data are means (standard deviations), unless otherwise indicated

*ART* antiretroviral therapy, *HAND* HIV-associated neurocognitive disorder

Significant differences (*p* < 0.045) between cognitively unimpaired patients and those with HAND were confined to NSS total scores and the subscores “orientation” and “complex motor tasks” with higher levels for patients with HAND (Table 2). NSS total scores were not significantly correlated with age or education (*p* > 0.09). Furthermore, no significant correlations between NSS scores and CD4 counts or any other clinical variables could be revealed (*p* > 0.09). Additionally, no significant differences (*p* > 0.15) emerged between HIV+ patients with and without lifetime history of major depressive disorder concerning NSS total scores and the respective subscores.

**Table 2:** NSS of the whole patient group and the respective subgroups, comparisons: cognitively unimpaired vs. cognitively impaired/HAND patients

	**Patients, whole group** **(*N* = 36)**	**Subgroups**		**Main effects, *T*-values**_**[df]**_
		Cognitively unimpaired patients (*N* = 16)	HAND patients (*N* = 20)	
NSS total score	9.81 (6.18)	7.50 (3.92)	11.65 (7.09)	*T*_[1, 34]_ = −2.096; *p* = 0.044
Motor coordination	2.64 (2.96)	2.00 (2.07)	3.15 (3.48)	*T*_[1, 34]_ = −1.165; *p* = 0.252
Orientation	1.17 (1.32)	0.56 (0.63)	1.65 (1.53)	*T*_[1, 26.377]_ = −2.886; *p* = 0.008
Sensory integration	2.19 (1.33)	2.06 (1.48)	2.30 (1.22)	*T*_[1, 34]_ = −0.528; *p* = 0.601
Complex motor tasks	1.64 (1.53)	1.00 (0.82)	2.15 (1.79)	*T*_[1, 27.826]_ = −2.565; *p* = 0.016
Hard signs	2.17 (1.84)	1.88 (1.78)	2.40 (1.90)	*T*_[1, 34]_ = −0.845; *p* = 0.404

Data are means (standard deviations)

### Volumetric correlates of NSS in patients with HIV

When cognitively unimpaired patients and patients with HAND were compared with respect to GM, the HAND subgroup showed significantly reduced GM in right superior temporal gyrus (BA 18; *k* = 164, *T* = 4.02, 36,8, − 26); however, this contrast was not confirmed after correction for multiple comparisons.

**Fig. 1:**
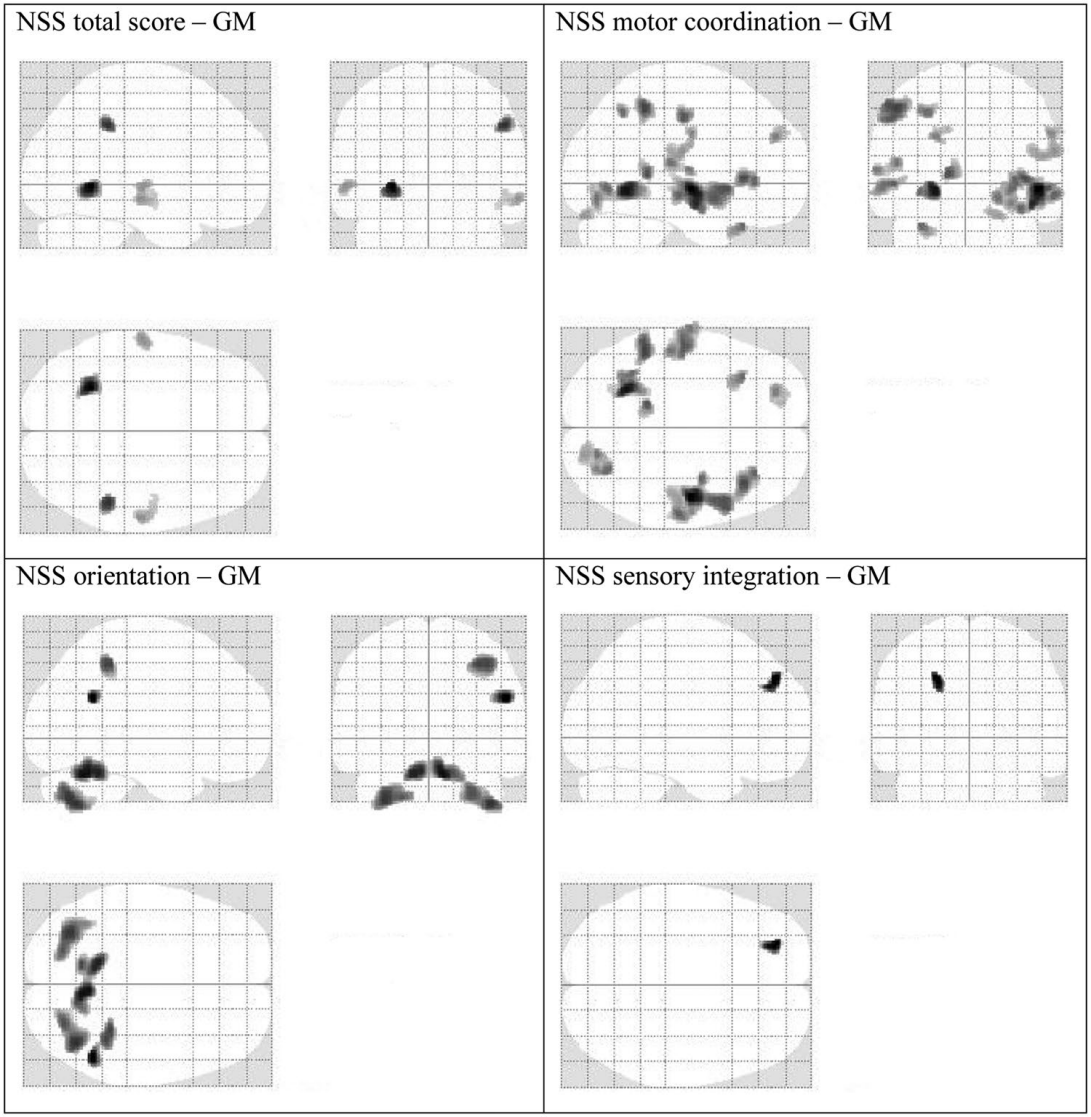
Brain regions showing significant inverse correlations between neurological soft signs (NSS) and gray matter (GM) in HIV+ patients with TIV as covariate (*p* < 0.001, uncorrected). Extent threshold *k* = 100 voxels

Higher NSS total scores were significantly correlated with reduced GM in left parahippocampal gyrus, right supramarginal gyrus, right middle temporal gyrus, and left superior temporal gyrus (Table 3, Fig. 1). However, none of these associations survived the FWE correction for multiple comparisons. The subscores “motor coordination”, “orientation”, and “sensory integration” showed significant correlations with GM in a variety of cerebral regions, with right insula and right cerebellum remaining significant after a stringent correction for multiple comparisons (*p* < 0.05). ﻿With respect to the subscales “complex motor tasks” and “hard signs”, no significant correlations with GM emerged. There were no significant positive correlations between NSS total scores and GM. When calculating Pearson’s correlation coefficients between absolute GM volumes and NSS scores, we obtained p-values of  >0.08. No significant correlations emerged between CD4 counts (actual CD4 count, CD4 nadir) or viral load at time of diagnosis and GM (*p* > 0.40).﻿

**Table 3:** Anatomical structures showing significant inverse correlations between neurological soft signs (NSS) and gray matter (GM) in HIV+ patients with TIV as covariate (*p* < 0.001, uncorrected)

	**Anatomical structures**	**Cluster size** **(voxel)**	***T*-value**	**Peak coordinates**^**1**^ **(*x*, *y*, *z*)**
NSS total score				
	Left limbic lobe, parahippocampal gyrus (BA 19)	312	5.18	−27, −54, −3
	Right parietal lobe, supramarginal gyrus (BA 40)	192	4.57	50, −41, 38
	Right temporal lobe, middle temporal gyrus (BA 21)	158	3.82	60, −20, −11
	Left temporal lobe, superior temporal gyrus (BA 22)	110	3.81	−59, −18, −3
Motor coordination				
	**Right sub-lobar, insula (BA 13)**^**a**^	**1428**	**5.16**	**48,** −**11,** −**6**
	Left limbic lobe, parahippocampal gyrus (BA 19)	519	4.91	−24, −57, −6
	Left parietal lobe, inferior parietal lobule (BA 40)	493	4.43	−50, −44, 48
	Right sub-lobar, insula (BA 13)	423	4.35	39, 24, 2
	Left limbic lobe, parahippocampal gyrus (BA 30)	183	4.30	−14, −42, 6
	Left parietal lobe, postcentral gyrus (BA 2)	270	4.26	−53, −17, 44
	Left temporal lobe, superior temporal gyrus	365	4.18	−53, −14, −2
	Right occipital lobe, fusiform gyrus (BA 19)	495	4.11	32, −71, −5
	Left frontal lobe, inferior frontal gyrus (BA 47)	146	4.10	−32, 24, −30
	Left parietal lobe, precuneus (BA 19)	122	4.08	−29, −60, 47
	Left temporal lobe, transverse temporal gyrus (BA 41)	234	4.04	−50, −26, 12
	Left frontal lobe, superior frontal gyrus (BA 9)	182	3.94	−21, 47, 29
	Right frontal lobe, precentral gyrus (BA 4)	375	3.87	57, −11, 32
	Right cerebellum, posterior lobe, uvula	123	3.74	21, −87, −23
Orientation				
	Right temporal lobesuperior temporal gyrus (BA 39)	136	4.62	51, −54, 26
	**Right cerebellum, anterior lobe nodule**^**b**^	1057	4.48	8, −60, −26
	**Right cerebellum, posterior lobe, cerebellar tonsil**^**c**^	871	4.22	41, −66, −45
	Left cerebellum, posterior lobe, inferior semi-lunar lobule	797	4.18	−32, −71, −42
	Right parietal lobe, inferior parietal lobule (BA 40)	348	4.10	39, −45, 48
Sensory integration				
	Left frontal lobe, middle frontal gyrus (BA 8)	131	4.28	−26, 47, 39

In bold: regions that survived a threshold of *p* ≤ 0.05 corrected for multiple comparisons

*BA* Brodmann area; extent threshold *k* = 100 voxels

^1^Talairach coordinates

^a^*p* = 0.006 FWE-corrected

^b^*p* = 0.026 FWE-corrected

^c^*p* = 0.050 FWE-corrected

## Discussion

In the present study, we sought to investigate NSS with respect to cerebral changes in HIV+ patients with and without evidence of neuropsychiatric deficits. While cognitively impaired patients with HAND had significantly elevated scores of total NSS and the subscores “orientation” and “complex motor tasks” in contrast to cognitively unimpaired patients, groups showed only minor differences with respect to GM in the right superior temporal gyrus. However, higher NSS scores were significantly correlated with reduced GM in a variety of cerebral sites. These correlations remained significant after FWE correction for multiple comparisons for right insula and cerebellum, an effect which applied to the NSS subscores “motor coordination” and “orientation”.

### NSS in patients with HIV

Consistent with earlier findings of Toro et al. (2018), we confirmed elevated NSS levels, i.e., NSS total score and subscores “orientation” and “complex motor tasks” in patients with HAND in comparison to cognitively healthy HIV+ patients. Similar elevations of NSS levels were corroborated in patients with chronic schizophrenia or patients with MCI and AD in contrast to healthy controls (Chrobak et al. 2016; Herold et al. 2018; Li et al. 2012; Schröder et al. 1992; Seidl et al. 2009; Urbanowitsch et al. 2015) that means in patient groups who typically show a severe neurocognitive involvement. In addition, it has been shown that in patients with chronic schizophrenia, bipolar disorder, AD/MCI, and healthy controls NSS are significantly associated with a wide range of cognitive domains, including processing speed, cognitive flexibility, working memory, episodic (autobiographical) memory, and theory of mind (Chan et al. 2011; Goswami et al. 2006; Herold et al. 2019; Li et al. 2012; Urbanowitsch et al. 2015). In schizophrenia, the clinical condition in which most studies focused on, NSS are considered a precursor of the manifestation of the disease, and, moreover, NSS vary with psychopathological symptoms during the course of the disorder (Bachmann et al. 2014; Bachmann and Schröder 2018). Therefore, from a clinical perspective, NSS may be used to identify individuals at risk to develop schizophrenia and patients at risk for a chronic course of the disease. These findings also apply to AD with MCI as its clinical precursor (Urbanowitsch et al. 2015) and HIV infections (Forno et al. 2020; Toro et al. 2018) and correspond to the transdiagnostic character of NSS.

In fact, already in the 1990s, sensitive motor tests were described as an indicator of subclinical lesions in HIV-infected patients, which precede structural brain alterations (Arendt et al. 1990). Improvement of motor performance in patients with HIV under therapy was reported by the same group, therefore pointing to a reliable therapy control measurement (Arendt et al. 1992). Moreover, early detectable motor impairment can be used as a predictor for HIV-related cerebral disease progression (Arendt et al. 1994). Recently, these results were confirmed and extended with the description of poorer neurocognitive performance in participants with greater motor dysfunction assessed using the HIV-Dementia Motor Scale (Elicer et al. 2018). Moreover, results showed that cognitive impairment is mild and stable over a period of at least 4 years in HIV+ patients treated with combined antiretroviral therapy, while motor function declines over time (*N* = 164, age 52 ± 9 years). This dissociation was explained by the high prevalence of cardiovascular comorbidities in their sample, a factor that was not given in our younger patient group. In contrast to the Heidelberg Scale, the HIV-Dementia Motor Scale additionally contains the components muscular strength, tone, and reflexes, while the former also includes sensory deficits.

Taken together, if replicated in larger studies, NSS may be considered a valid and easy to administer clinical marker of severity and maybe course of these neuropsychiatric disorders in settings without access to detailed neuropsychological testing. More specifically, given the lack of a validated screening instrument especially for milder HAND conditions (Barber et al. 2014; Zipursky et al. 2013) and the time and experience necessary for thorough neuropsychological testing (Robertson et al. 2016), NSS could be a useful tool for the identification of early cognitive deficits in patients with HIV. In general, the importance of motor deficits in HAND is underlined by its diagnostic criteria, which include — besides typical neuropsychological deficits in attention/working memory, executive functions, memory, verbal abilities or information processing speed — also impaired motor skills (Antinori et al. 2007; Woods et al. 2009). However, our findings need to be replicated, especially with respect to sensitivity and specificity for classification of individual patients.

In the present study, NSS total scores were not significantly correlated with age, which is consistent with the results of a previous study of our group (Urbanowitsch et al. 2015) referring to two birth cohorts of healthy controls (born 1930–1932 and 1950–1952). Given a relatively young and homogeneous group as in the present study, age-related effects of NSS cannot be expected.

In contrast to the former results of our group (Herold et al. 2018; Urbanowitsch et al. 2015), we could not confirm a significant association between NSS total scores and years of education. The sample of the present study consists of rather young people with HIV infection recruited from an outpatient infectious disease clinic, which may explain their high level of education, a factor that limits the generalizability of our results. Indeed, the level of education in our patient group is slightly higher than in other clinical groups in Chile (Musa et al. 2017; Slachevsky et al. 2019) but corresponds to that of HIV+ patients from the same outpatient infectious disease clinic (Ceballos et al. 2016). Moreover, the recruitment of our patients may also have contributed to the rather high percentage of patients with HAND of 56%, which is, nevertheless, in the range reported in other studies (Saloner and Cysique 2017; Smith and Sacktor 2014). However, population-specific cognitive test norms were not available, except for verbal fluency (Guàrdia-Olmos et al. 2015), but *z*-values were corrected not only for age and sex (using normative data of the CANTAB), but also for verbal IQ, which is validated for the Chilean population (Rosas et al. 2014).

As in patients with HIV the prevalence of major psychiatric conditions is higher than in the general population (Ciesla and Roberts 2001; Owe-Larsson et al. 2009), an exclusion of those with any concomitant psychiatric disease would have resulted in selection effects. Therefore, 18 patients with lifetime history of major depressive disorder, 8 with current depression and 5 patients with substance abuse in the past, were included in our sample. However, no significant differences emerged between patients with and without lifetime history of major depressive disorder referring to NSS total score and the respective subscores. This is supported by the results of a study of Zhao et al. (2013), who found comparable NSS scores in patients with major depression and healthy controls.

### Volumetric correlates of NSS in patients with HIV

MRI studies have been used since the early times to examine the impact of the virus on the central nervous system and revealed gray matter atrophy especially affecting the basal ganglia, lesions of white matter, and ventricular enlargement/global cerebral atrophy. The question of the involvement of the central nervous system remains important as in the era of modern ART HIV-associated dementia is becoming less frequent, while an increase of less severe cognitive symptoms is noticeable (Tate et al. 2011).

The comparison of cognitively unimpaired patients and patients with HAND concerning GM revealed in the HAND subgroup significantly reduced GM of right superior temporal gyrus; however, this difference did not survive the correction for multiple comparisons. In these milder stages of ANI and MND neuropsychological deficits may reflect rather functional impairments with correlations on a structural level in later, more severe forms as HIV-associated dementia (Arendt et al. 1990). On the other side, one can speculate that NSS are a more sensitive indicator of neuropsychological deficits than structural changes, an assumption that is supported by a wide range of cognitive domains associated with NSS (Chan et al. 2011; Forno et al. 2020; Goswami et al. 2006; Herold et al. 2019; Urbanowitsch et al. 2015).

Our finding of only minor GM differences between cognitively unimpaired patients and patients with HAND is well in-line with a recent publication by Heaps et al. (2015). Thirty-seven HIV+ patients with HAND and 37 HIV+ patients with normal cognitive function, both treatment-naïve, and 29 HIV-uninfected controls were examined with structural MRI in Thailand. While HIV+ patients with HAND showed significantly smaller brain volumes in subcortical and total GM in comparison to the uninfected controls, no statistically significant differences emerged between HIV+ patients with and without HAND. Moreover, no significant volumetric differences emerged between the HIV+ groups categorized by functional impairment, which means cognitively symptomatic (MND, dementia) versus asymptomatic patients (ANI, HIV+ patients with normal cognitive function).

Similarly, values of diffusion tensor imaging could not significantly differ between HIV+ patients with (*N* = 10) and without HAND (*N* = 12). In a ROI-based approach, however, HIV+ patients with HAND showed altered microstructures in the right superior longitudinal fasciculus in comparison to HIV+ patients without HAND (Oh et al. 2018). Together with the results of another study using diffusion tensor imaging (Zhu et al. 2013), we conclude that these microstructural alterations differentiating HIV+ patients with and without HAND may not be detectable in GM volume as in our study.

Structural neuroimaging studies about the cerebral correlates of NSS in patients with schizophrenia reported the pre- and postcentral gyri, premotor area, cerebellum, middle and inferior frontal gyri, thalamus and basal ganglia, temporal and lingual gyri, inferior parietal lobule, insula, precuneus, and occipital gyrus as important sites of NSS (Hirjak et al. 2015; Zhao et al. 2014). These results were confirmed and extended to a group of HIV+ patients, which also showed significant negative associations between NSS scores and GM in pre- and postcentral gyri, cerebellum, middle and inferior frontal gyri, temporal gyri, inferior parietal lobe, insula, and occipital lobe. However, we could not confirm a contribution of the thalamus and basal ganglia. Indeed, we recently described that region of interest analyses are especially useful — in contrast to a whole brain approach — in showing that these smaller subcortical structures are the most affected by motor NSS in a sample of 81 first-episode psychosis patients (Quispe Escudero et al. 2020).

After correction for multiple comparisons, two areas remained significant in the present study: right insula and right cerebellum.

The NSS subscale “orientation” correlated significantly with reduced volume of the right cerebellum (anterior and posterior lobe). This result underlines the important role of the cerebellum in sensorimotor control, motor learning, and motor coordination (Ito 1972; Manto et al. 2012; Phillips et al. 2015). Especially, its function in coordinating and monitoring the acquisition of sensory information can explain the associations with the NSS subscale “orientation” consisting of the items right/left orientation, graphesthesia, face-hand test, and stereognosis (Manto et al. 2012; Rondi-Reig et al. 2014). Besides volumetric reduction in cortical regions, which are involved in motor processes as primary motor, sensory, supplementary motor, and premotor cortices, also basal ganglia and cerebellum are affected in patients with HIV (for overview, see Zhou et al. 2017). However, the impact of these motor-associated brain structural impairments on the respective functions is not yet clear.

The NSS subscale “motor coordination” showed a significant inverse correlation with right insula (BA 13). The insular cortex has been shown to play an integrative role linking information from different functional systems (Chang et al. 2013; Mazzola et al. 2019). Moreover, a differentiation of the insular cortex into four functional regions has been revealed meta-analytically (Kurth et al. 2010): a sensorimotor, a cognitive, a social-emotional, and an olfacto-gustatory domain were defined. A recent study demonstrated — paralleling our results — a reduction of GM and abnormal activation during motor performance in 22 treatment-naive patients with HIV infection (Zhou et al. 2017). Patients showed significantly reduced GM in cortical regions involved in motor control (including bilateral posterior insula, premotor cortex, and supramarginal gyrus). Besides this, core nodes of cognitive processing networks (anterior cingulate cortex, left dorsolateral prefrontal cortex, superior temporal gyrus, inferior temporal cortex, medial orbito-frontal cortex) were also affected. Compared with healthy controls, patients had an increased activation in bilateral posterior insula during hand movement tasks. Thus, the GM reduction in bilateral posterior insula cortices was spatially coincident with abnormal brain activation in the patient group. It can be speculated that the bilateral insula showed greater activation during the performance of the hand movement task to compensate for potential deficits in patients with HIV infection.

Taken together, our finding of associations between insula and cerebellum with NSS performance confirms at least partly the assumption of a sensorimotor network as conceptualized by Habas et al. (2009), which includes, among others, insula and cerebellum.

In summary, our results underline that NSS in patients with HIV can be used as a marker of cerebral damage and neurocognitive deficits and therefore may facilitate the early recognition of HAND especially in resource-limited settings. The neurocognitive aspects of HIV infection are especially important, as, due to modern antiretroviral treatment, an increasing age of infected people can be observed together with HAND in earlier phases of the disease (Eggers et al. 2017; Ellis et al. 2009). Furthermore, the reported cerebral correlates of NSS in HIV+ patients with and without HAND may contribute to a better understanding of the underlying mechanisms. However, given a relatively small sample size and an exploratory approach, our findings need to be replicated and may stimulate further research in this field. Especially, other neuroimaging and longitudinal studies in patients with HIV are necessary to learn more about the course of NSS during HIV infection and to establish their prognostic value.

## Footnotes

1 Most rapid voluntary alternating index finger movements and rise time of most rapid index finger extensions.

2 14 parameters: speech, facial expression, tremor at rest, action or postural tremor of hands, rigidity, arise from chair, posture, postural stability bradykinesia, finger tapping, hand opening/closing, hand pronation/supination, heel tapping, gait, body bradykinesia.

3 HIV-Dementia Motor Scale: 5 motor domains (strength, tone, reflexes, coordination, and gait).

4 Modified HIV Motor Scale: 5 subscores (gait, coordination, strength, tone, reflexes).

5 A detailed manual is available in English, German, and Spanish on request.

6 http://www.fil.ion.ucl.ac.uk/spm/software/

7 http://www.neuro.uni-jena.de/cat/

8 http://www.brainmap.org/ale/

9 http://www.talairach.org/client.html
